# Mitogenomic phylogenies support the validity of the family Micracanthorhynchinidae (Acanthocephala: Echinorhynchida), with novel gene arrangement in the mitogenomes of *Micracanthorhynchina hemirhamphi* and *Rhadinorhynchus laterospinosus*

**DOI:** 10.1186/s13071-025-06972-z

**Published:** 2025-08-03

**Authors:** Yuan-Yuan Xie, Ke-Yu Wang, Rui-Jia Yang, Hui-Xia Chen, Li Liang

**Affiliations:** 1 Hebei Collaborative Innovation Center for Eco‐Environment; Hebei Key Laboratory of Animal Physiology, Biochemistry and Molecular Biology; College of Life Sciences, Hebei Normal University, Shijiazhuang, 050024 Hebei Province People’s Republic of China; 2 Ministry of Education Key Laboratory of Molecular and Cellular Biology, Shijiazhuang, 050024 Hebei Province People’s Republic of China

**Keywords:** Parasite, Acanthocephalan, Rhadinorhynchidae, Mitogenome, Micracanthorhynchinidae, Phylogeny

## Abstract

**Background:**

The family Rhadinorhynchidae is a common group of acanthocephalans parasitizing various marine and freshwater fishes. The current knowledge of the pattern of mitogenomic evolution of the rhadinorhynchid acanthocephalans is still extremely limited. The monophyly of the Rhadinorhynchidae and the phylogenetic status of several of its included genera and subfamilies remain under debate.

**Methods:**

The complete mitogenomes of *Micracanthorhynchina hemirhamphi* and *Rhadinorhynchus laterospinosus* were sequenced and annotated for the first time on the basis of the specimens collected from the Asian pencil halfbeak *Hyporhamphus intermedius* (Cantor) (Beloniformes: Hemiramphidae) and the frigate tuna *Auxis thazard* (Lacepède) (Scombriformes: Scombridae), respectively. Phylogenetic analyses of Acanthocephala were performed on the basis of the concatenated amino acid sequences of 12 protein-coding genes (PCGs) of mitogenomes using maximum likelihood (ML) and Bayesian inference (BI), respectively.

**Results:**

The complete mitogenomes of *M. hemirhamphi* and *R. laterospinosus* are 17,272 bp and 13,567 bp in length, which both include 36 genes, containing 12 PCGs (missing *atp8*), 22 transfer RNA (tRNA) genes, and two ribosomal RNAs (*rrnS* and *rrnL*), plus two noncoding regions. Additionally, several tRNA gene rearrangement events occurred in the mitogenomes of both *M. hemirhamphi* and *R. laterospinosus*. Phylogenetic results supported the traditional rhadinorhynchid genus *Micracanthorhynchina* as a distinct lineage from Rhadinorhynchidae and Cavisomatidae.

**Conclusions:**

The mitogenome of *M. hemirhamphi* represents the largest mitogenome of acanthocephalan reported so far. The mitogenome of *R. laterospinosus* is the smallest mitogenome of the order Echinorhynchida, which also represents the first mitogenomic data for the genus *Rhadinorhynchus* and also for the Rhadinorhynchidae sensu stricto. Comparative mitogenomic analyses revealed the gene arrangements of *R. laterospinosus* and *M. hemirhamphi* represent two new types of mitochondrial gene arrangement reported in Acanthocephala. Moreover, mitogenomic phylogenies further confirmed the validity of the family Micracanthorhynchinidae and suggested a sister relationship Micracanthorhynchinidae + (Rhadinorhynchidae + Cavisomatidae) within Echinorhynchida.

**Graphical Abstract:**

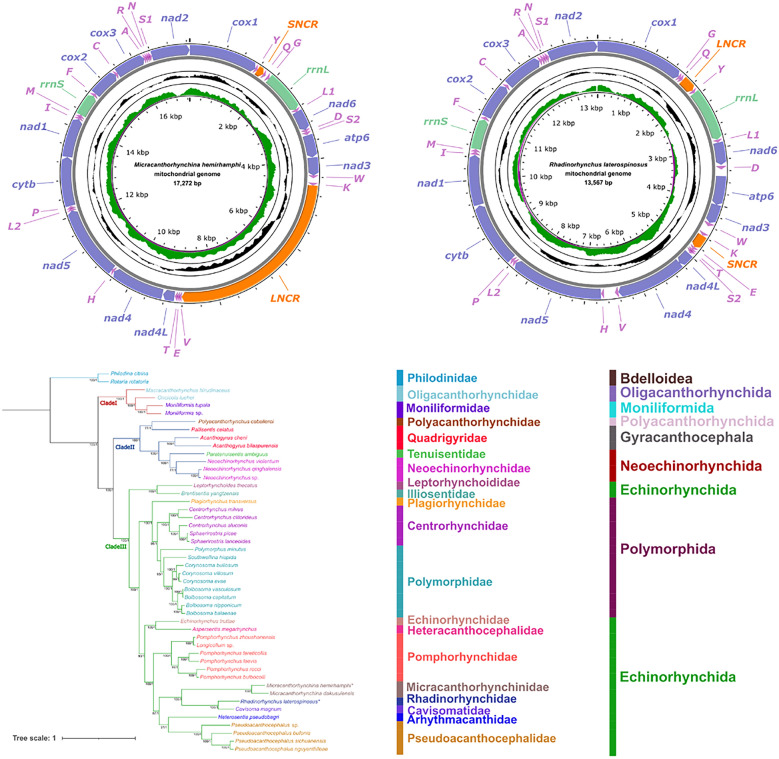

Created in BioRender. Hardy, H. (2025) https://BioRender.com/mo3g1wr

**Supplementary Information:**

The online version contains supplementary material available at 10.1186/s13071-025-06972-z.

## Background

The Acanthocephala is an important phylum of endoparasites, occurring in the alimentary canal of various vertebrates globally, which are of veterinary, medical, and economic importance [1–3]. The family Rhadinorhynchidae is a diverse group of acanthocephalans, currently comprising more than 120 species parasitizing various marine and freshwater fishes worldwide [2, 4]. According to the traditional classification [2], the Rhadinorhynchidae includes 24 genera assigned into five subfamilies, namely Golvanacanthinae, Gorgorhynchinae, Rhadinorhynchinae, Serrasentinae, and Serrasentoidinae. However, the validity and systematic status of several of its included genera and subfamilies were challenged by the molecular phylogenetic studies [4–10]. The monophyly of the Rhadinorhynchidae, and the phylogenetic relationships of Rhadinorhynchidae and the other families within the order Echinorhynchida have long been controversial [4, 9, 10].

Steinauer et al. [11] reported the first mitochondrial genome of acanthocephalan species (*Leptorhynchoides thecatus*). Since then, numerous acanthocephalan mitogenomes have been sequenced [12–25]. Meanwhile, mitogenome-based phylogenetic analyses of Acanthocephala are increasingly popular, which challenged and improved the traditional classifications of Acanthocephala, and also enable a better understanding of acanthocephalan evolution [12–25]. However, the current mitogenomic data of Acanthocephala are still limited, and the present knowledge of the characteristics of acanthocephalan mitogenomes remains far from complete. In the Rhadinorhynchidae, only *Micracanthorhynchina dakusuiensis* has had a full mitogenome sequenced and reported [26].

The genus *Micracanthorhynchina*, with only 12 nominal species parasitic in fishes, is a small group of acanthocephalans in the Rhadinorhynchidae [2, 27, 28]. Yamaguti erected the family Micracanthorhynchinidae and designed *Micracanthorhynchina* as the type genus [1]. The Micracanthorhynchinidae originally included five genera, namely *Cleaveius*, *Hemirhadinorhynchus*, *Micracanthorhynchina*, *Pseudauchen*, and *Pseudorhadinorhynchus*. However, the validity of the Micracanthorhynchinidae was rejected by the subsequent taxonomists, which was treated as a synonym of the family Rhadinorhynchidae [2, 29]. The genus *Micracanthorhynchina* was also placed into the subfamily Gorgorhynchinae of the Rhadinorhynchidae [2]. Yet, several recent molecular phylogenetic analyses based on the 18S + *cox1* or 18S + 28S + *cox1* sequence data questioned the current systematic position of *Micracanthorhynchina* within the Rhadinorhynchidae [10, 28] and suggested the resurrection of the validity of the family Micracanthorhynchinidae [10].

In the present study, to enrich the mitogenome data of Acanthocephala and further reveal the evolutionary pattern of mitogenomes of the order Echinorhynchida, the complete mitogenomes of *Micracanthorhynchina hemirhamphi* and *Rhadinorhynchus laterospinosus* were sequenced and annotated for the first time. Furthermore, to investigate the phylogenetic relationships of different families within the order Echinorhynchida, with emphasis on the Micracanthorhynchinidae and Rhadinorhynchidae, phylogenetic analyses based on the concatenated amino acid sequences of 12 protein-coding genes (PCGs) of mitogenomes of Acanthocephala were performed using maximum likelihood (ML) and Bayesian inference (BI), respectively.

## Methods

### Parasite collection and species identification

**Fig. 1:**
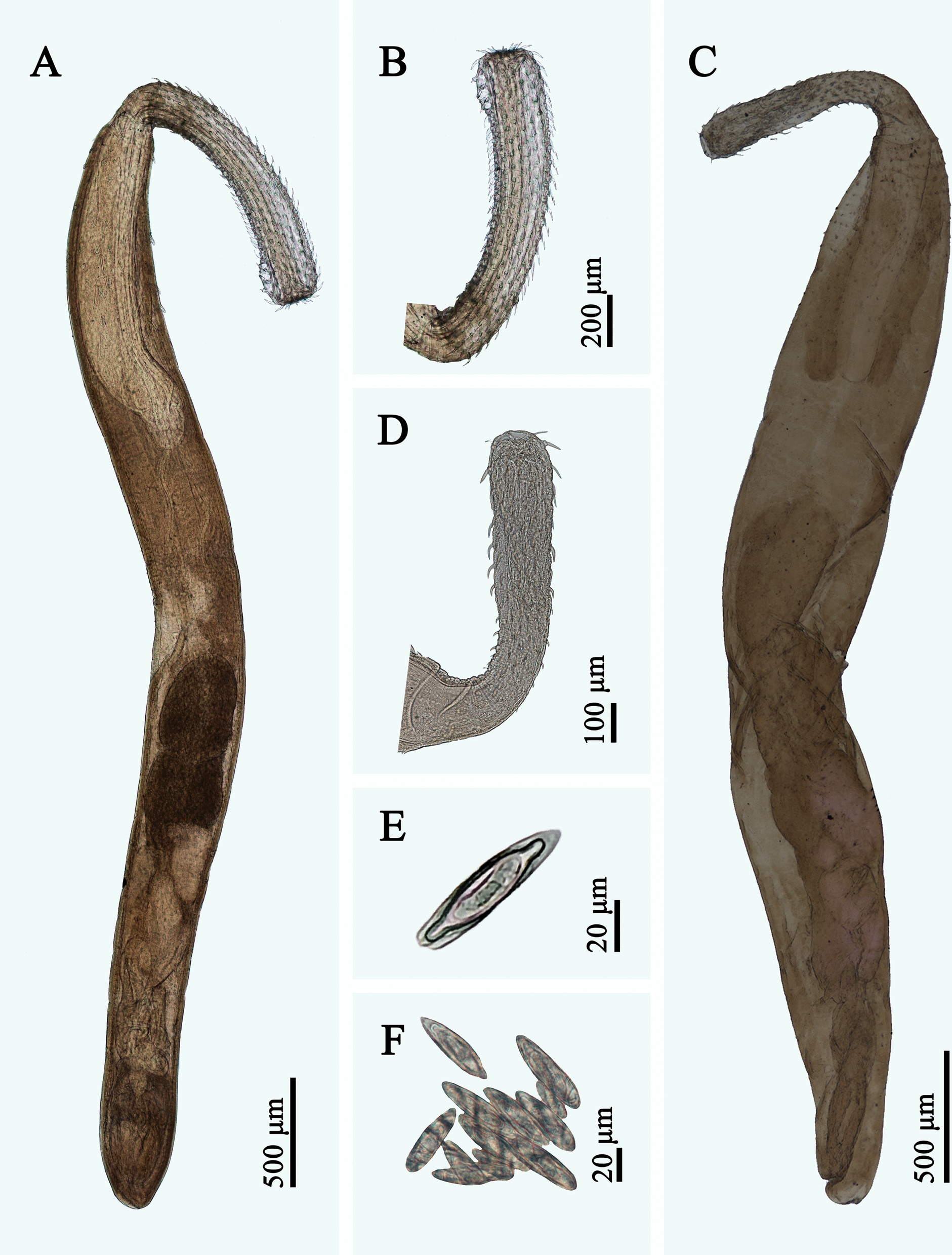
Photomicrographs of *Micracanthorhynchina hemirhamphi* and *Rhadinorhynchus laterospinosus* collected from *Hyporhamphus intermedius* and *Auxis thazard*. **A**, mature male of *R. laterospinosus*, **B**, proboscis of *R. laterospinosus*, **C**, mature male of *M. hemirhamphi*, **D**, proboscis of *M. hemirhamphi*, **E**, egg of *R. laterospinosus*, **F**, eggs of *M. hemirhamphi*

During a helminthological survey of Chinese marine fishes in the South China Sea, the acanthocephalans of *M. hemirhamphi* and *R. laterospinosus* were collected from the intestine of the Asian pencil halfbeak *Hyporhamphus intermedius* (Cantor) (Beloniformes: Hemiramphidae) (off Qinzhou, Guangxi Zhuang Autonomous Region, China) and the frigate tuna *Auxis thazard* (Lacepède) (Scombriformes: Scombridae) (off Shanwei, Guangdong Province, China), respectively. The specimens were washed in tap water and preserved in 80% ethanol for further study. Acanthocephalans were identified to species level based on the morphological features and genetic data reported in some previous studies [28, 30–32]. The morphometric comparisons and photomicrographs of *M. hemirhamphi* and *R. laterospinosus* are provided in Fig. 1 and Tables 1 and 2. Voucher specimens of *M. hemirhamphi* (HBNU–A–F2024060WL) and *R. laterospinosus* (HBNU–A–F2024061ZL) were deposited in the College of Life Sciences, Hebei Normal University, China.

**Table 1:** Morphometric comparisons of *Micracanthorhynchina hemirhamphi*

Source	Present study		Wang, 1991		Baylis, 1944	
Host	*Hemiramphus intermedius*		*Hemiramphus intermedius*, *Hyorhamphus sajori*		*Hemiramphus intermedius*	
Locality	South China Sea		Taiwan Strait		Off New Zealand	
Characteristics	Male	Female	Male	Female	Male	Female
TL	3.80–4.27	5.90	2.80–4.00	5.60–6.20	3.00–4.30	4.50–6.50
TW	0.59–0.66	0.85	0.45–0.62	0.95–0.98	0.46–0.67	0.80–0.97
SP	0.59–0.83 × 0.14–0.16	0.85 × 0.16	0.56 × 0.16	0.86–0.92 × 0.21	0.55 × 0.16–0.17	0.55–0.65 × 0.18–0.20
NRP	12	12	12	12	12	12
NHPR	10–11	10–11	10–11	10–11	9–11	9–11
NTRS	14–22	14–22	14–22	14–22	15–17	15–17
SPR	1.02–1.17 × 0.11–0.20	1.49 × 0.23	0.80 × 0.16	1.12 × 0.29	0.8–0.9 × 0.15	0.90–1.10 × 0.17–0.20
LL	0.93–1.05	1.63–1.65	0.80	0.96–1.20	–	–
SAT	0.61–0.65 × 0.25–0.34	N/A	–	N/A	–	N/A
SPT	0.55–0.61 × 0.30–0.38	N/A	–	N/A	–	N/A
NCG	4	N/A	4	N/A	4	N/A
CA	N/A	Present	N/A	–	N/A	Present
SE	N/A	0.054–0.062 × 0.017–0.020	N/A	0.052–0.060 × 0.016–0.018	N/A	0.056–0.062 × 0.015–0.016

All measurements are in millimeters. TL, trunk length; TW, trunk width; SP, size of proboscis; NRP, number of longitudinal rows of proboscis hooks; NHPR, number of hooks per longitudinal row; NTRS, number of transverse rows of trunk spines; SPR, size of proboscis receptacle; LL, length of lemnisci; SAT, size of anterior testis; SPT, size of posterior testis; NCG, number of cement glands; CA, caudal appendage in female; SE, size of eggs

**Table 2:** Morphometric comparisons of *Rhadinorhynchus laterospinosus*

Source	Present study		Amin et al., 2011	Amin et al., 2019		Lisitsyna et al., 2023		Kita et al., 2024	
Host	*Auxis thazard*		*Balistes* sp.	*Alectis ciliaris*, *Auxis rochei*, *Auxis thazard*, *Leiognathus equulus*, *Lutjanus bitaeniatus*, *Megalaspis cordyla*, *Nuchequula flavaxilla*, *Tylosurus* sp.		*Katsuwonus pelamis*, *Scomber australasicus*, *Trichiurus lepturus*		*Auxis thazard*	
Locality	South China Sea		Off Vietnam	Off Vietnam		Taiwan Strait		Off Japan	
Characteristics	Male	Female	Female	Male	Female	Male	Female	Male	Female
TL	5.03–10.6	7.78–10.2	7.82	4.75–11.3	7.80–21.3	7.16–7.43	9.43–19.1	8.31–9.32	11.7–20.6
TW	0.30–1.08	0.43–0.55	0.41	0.35–0.80	0.35–1.00	0.49–0.62	0.53–0.69	0.62–0.81	0.54–0.87
SP	1.30–1.52 × 0.15–0.26	1.44–1.95 × 0.26–0.30	1.62 × 0.27	1.00–1.67 × 0.17–0.23	1.25–1.90 × 0.17–0.30	1.41–1.52 × 0.16–0.33	1.58–1.69 × 0.22–0.31	1.51–2.24 × 0.15–0.30	1.19–1.97 × 0.15–0.31
NPR	16	16	18	15–19	15–19	18	18	15–16	15–16
NHPR	21–25	23–25	24	21–26	21–26	21–22	26–28	22–24	22–24
SPR	1.48–2.93 × 0.20–0.38	1.45–4.10 × 0.30–0.38	–	1.62–3.45 × 0.14–0.35	2.24–3.95 × 0.17–0.37	1.74–2.09 × 0.14–0.30	2.48–3.83 × 0.16–0.35	1.97–3.20 × 0.29–0.43	2.77–4.62 × 0.18–0.33
LL	1.55–2.55	1.53–3.78	–	1.50–2.50	2.29–3.64	1.91–2.30	3.30–4.25	–	–
SAT	0.52–1.30 × 0.32–0.63	N/A	N/A	0.59–1.75 × 0.22–0.52	N/A	0.83–1.02 × 0.34–0.39	N/A	1.05–1.15 × 0.37–0.59	N/A
SPT	0.50–1.30 × 0.37–0.63	N/A	N/A	0.47–1.50 × 0.22–0.57	N/A	0.70–0.92 × 0.23–0.38	N/A	0.91–1.31 × 0.40–0.59	N/A
NCG	4	N/A	N/A	4	N/A	4	N/A	4	N/A
SE	N/A	0.028–0.033 × 0.009	0.062 × 0.017	N/A	0.057–0.068 × 0.012–0.018	N/A	0.083–0.085 × 0.016–0.019	N/A	–

All measurements are in millimeters. TL, trunk length; TW, trunk width; SP, size of proboscis; NRP, number of longitudinal rows of proboscis hooks; NHPR, number of hooks per longitudinal row; SPR, size of proboscis receptacle; LL, length of lemnisci; SAT, size of anterior testis; SPT, size of posterior testis; NCG, number of cement glands; SE, size of eggs

### Mitochondrial genome sequencing, assembly, and annotation

The genomic DNA of acanthocephalans was extracted using the Magnetic Universal Genomic DNA Kit (DP705) [Sangon Biotech (Shanghai) Co., Ltd., Shanghai, China] according to the manufacturer’s instructions, which was sent to Novogene (Tianjin, China) for constructing genomic DNA libraries following the company’s standard protocol. A total of 50 GB of clean data for each sample of *M. hemirhamphi* and *R. laterospinosus* were produced using the Pair-End 150 sequencing method on the Illumina NovaSeq 6000 platform by Novogene (Tianjin, China). The protocol and procedure for assembly and annotation of the complete mitochondrial genomes of *M. hemirhamphi* and *R. laterospinosus* are according to the previous studies [21–25]. The complete mitochondrial genomes of *M. hemirhamphi* and *R.* *laterospinosus* obtained herein are deposited in the GenBank database (http://www.ncbi.nlm.nih.gov) under the accession numbers: PV590120 (*M. hemirhamphi*) and PV590110 (*R. laterospinosus*).

### Phylogenetic analyses

Phylogenetic analyses were conducted based on the concatenated amino acid (AA) sequences of the 12 PCGs using maximum likelihood (ML) and Bayesian inference (BI) methods, respectively. *Rotaria rotatoria* and *Philodina citrina* (Rotifera: Bdelloidea) were treated as the out-group. The in-group included 44 acanthocephalan species belonging to six orders. Detailed information on the representatives of Acanthocephala included in the present phylogenetic analyses was provided in Table 3.

**Table 3:** Detailed information on representatives of Acanthocephala included in the present phylogeny

Taxa	Order	Family	Species	Accession	Size (bp)	AT%	References
*Outgroup*							
Rotifera	Bdelloidea	Philodinidae	*Rotaria rotatoria*	NC_013568	15,319	73.2	[33]
			*Philodina citrina*	FR856884	14,003	77.7	[34]
Ingroup							
Archiacanthocephala	Moniliformida	Moniliformidae	*Moniliformis* sp.	OK415026	14,066	66.2	[35]
			*Moniliformis tupaia*	OP413683	14,150	63.8	Unpublished
	Oligacanthorhynchida	Oligacanthorhynchidae	*Macracanthorhynchus hirudinaceus*	NC_019808	14,282	65.2	[34]
			*Oncicola luehei*	NC_016754	14,281	60.2	[12]
Eoacanthocephala	Gyracanthocephala	Quadrigyridae	*Acanthogyrus cheni*	KX108947	13,695	65.3	[36]
			*Acanthogyrus bilaspurensis*	MT476589	13,360	59.3	[20]
			*Pallisentis celatus*	NC_022921	13,855	61.5	[37]
	Neoechinorhynchida	Neoechinorhynchidae	*Neoechinorhynchus violentum*	KC415004	13,393	59.4	[38]
			*Neoechinorhynchus qinghaiensis*	MW851291	13,271	65.8	Unpublished
			*Neoechinorhynchus* sp.	MT345686	13,269	65.1	Unpublished
		Tenuisentidae	*Paratenuisentis ambiguus*	NC_019807	13,574	66.9	[34]
Palaeacanthocephala	Echinorhynchida	Arhythmacanthidae	*Heterosentis pseudobagri*	OP278658	13,742	62.5	[39]
		Cavisomidae	*Cavisoma magnum*	MN562586	13,594	63.0	[17]
		Echinorhynchidae	*Echinorhynchus truttae*	NC_019805	13,659	63.1	[34]
		Heteracanthocephalidae	*Aspersentis megarhynchus*	PP965112	14,661	64.6	[24]
		Pomphorhynchidae	*Longicollum* sp.	OR215045	14,632	55.8	Unpublished
			*Pomphorhynchus bulbocolli*	JQ824371	13,915	59.9	Unpublished
			*Pomphorhynchus laevis*	MN562482	13,881	57.5	[40]
			*Pomphorhynchus rocci*	JQ824373	13,845	60.7	Unpublished
			*Pomphorhynchus tereticollis*	JQ809452	13,890	56.8	Unpublished
			*Pomphorhynchus zhoushanensis*	MN602447	14,632	55.8	Unpublished
		Pseudoacanthocephalidae	*Pseudoacanthocephalus bufonis*	MZ958236	14,056	58.4	[21]
			*Pseudoacanthocephalus* sp.	OQ588705	14,883	61.5	Unpublished
			*Pseudoacanthocephalus sichuanensis*	PP476191	15,812	56.8	[22]
			*Pseudoacanthocephalus nguyenthileae*	PP476192	13,701	56.3	[22]
		Micracanthorhynchinidae	*Micracanthorhynchina dakusuiensis*	OP131911	16,309	56.8	[26]
			*Micracanthorhynchina hemirhamphi*	PV590120	17,272	60.5	Present study
		Rhadinorhynchidae	*Rhadinorhynchus laterospinosus*	PV590110	13,567	62.9	Present study
		Leptorhynchoididae	*Leptorhynchoides thecatus*	NC_006892	13,888	71.4	[11]
		llliosentidae	*Brentisentis yangtzensis*	MK651258	13,864	68.3	[41]
	Polymorphida	Centrorhynchidae	*Centrorhynchus clitorideus*	MT113355	15,884	55.5	[19]
			*Centrorhynchus milvus*	MK922344	14,314	54.5	[16]
			*Centrorhynchus aluconis*	KT592357	15,144	54.5	[14]
			*Sphaerirostris lanceoides*	MT476588	13,478	58.0	[18]
			*Sphaerirostris picae*	MK471355	15,170	58.1	[15]
		Polymorphidae	*Polymorphus minutus*	MN646175	14,149	64.4	[42]
			*Southwellina hispida*	NC_026516	14,742	63.9	[13]
			*Bolbosoma balaenae*	MZ357084	14,301	62.6	[43]
			*Bolbosoma capitatum*	MZ357085	14,319	63.9	[43]
			*Bolbosoma vasculosum*	MZ357087	14,313	63.9	[43]
			*Bolbosoma nipponicum*	OR468096	14,296	60.9	[23]
			*Corynosoma villosum*	OR468095	14,241	61.0	[23]
			*Corynosoma bullosum*	PQ516697	14,879	63.8	[25]
			*Corynosoma evae*	PQ516696	13,947	61.6	[25]
		Plagiorhynchidae	*Plagiorhynchus transversus*	NC_029767	15,477	61.1	[14]
Polyacanthocephala	Polyacanthorhynchida	Polyacanthorhynchidae	*Polyacanthorhynchus caballeroi*	NC_029766	13,956	56.3	[14]

Amino acid sequences were extracted and aligned individually using the E-INS-i strategy under MAFFT v7.313 [44] then concatenated into a single alignment matrix using PhyloSuite v1.2.2 [45]. The best-fitting substitution model Jones + I + G + F was selected for Bayesian inference using ModelFinder [46], according to the Bayesian information criterion (BIC). The Bayesian inference was performed for 5 × 10^6^ MCMC generations under MrBayes v3.2.

IQ-TREE v2.1.2 was used for the maximum likelihood (ML) inference. The best-fitting substitution model mtZOA + F + I + G4 was selected by ModelFinder [46], according to the Akaike information criterion (AIC). Nodal support for the ML tree was evaluated using 1,000 bootstrap pseudoreplicates with the ultrafast bootstrap approximation method [47], while other parameters were maintained at default settings [48, 49]. Phylogenetic trees were visualized using iTOL v6.1.1 [50].

## Results

### Species identification

The morphology and morphometric data of the present specimens of *M. hemirhamphi* are almost identical to the previous descriptions of this species [30, 53] regarding several features, including the size of trunk and proboscis receptacle, the shape (size) and armature of the proboscis, the number of transverse rows of trunk spines, the length of lemnisci, the shape and length of testis, the number of cement glands, the size of eggs, and the presence of caudal appendage in female (see Table 1 for details). Moreover, the present specimens were also collected from the type host of *M. hemirhamphi*, *Hemiramphus intermedius* (Cantor) (Beloniformes: Hemiramphidae). Therefore, we consider that the present specimens are conspecific with *M. hemirhamphi*. However, there has been no genetic data of *M. hemirhamphi* available on the GenBank database; thus, we could not further identify these specimens using the molecular-based method.

The morphology and morphometric data of the present specimens of *R. laterospinosus* agreed well with the previous descriptions of this species [10, 28, 31, 32] regarding several features, including the size of trunk and proboscis receptacle, the shape (size) and armature of the proboscis, the length of lemnisci, the shape and length of testis, the number of cement glands, and the size of eggs (see Table 2 for details). Additionally, comparison of the *cox1* data of the present material and *R. laterospinosus* available in GenBank (MK572741-MK572744, LC777823, OR625530, and OR625531) showed a very high level of similarity (97.9–98.9%). Consequently, we considered our material belonging to *R. laterospinosus*.

### Characterization of the complete mitogenomes of M. hemirhamphi and R. laterospinosus

**Fig. 2:**
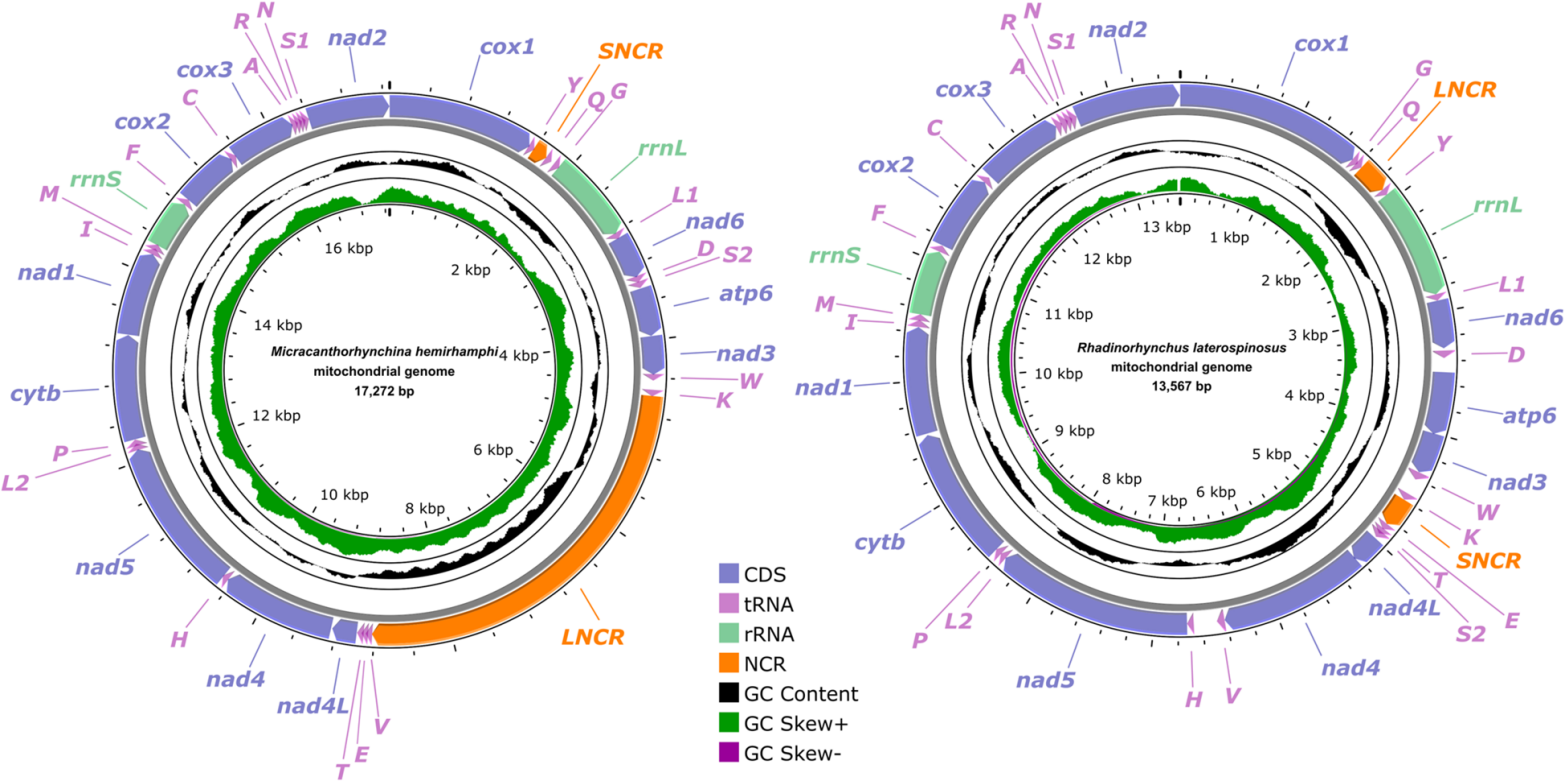
Gene map of mitochondrial genomes of *Micracanthorhynchina hemirhamphi* and *Rhadinorhynchus laterospinosus*. All 22 tRNA genes are nominated by the one-letter code with numbers differentiating each of the two tRNAs serine and leucine. All genes are transcribed in the clockwise direction on the same strand. The outermost circle shows the GC content, and the innermost circle shows the GC skew

The complete mitogenomes of *M. hemirhamphi* and *R. laterospinosus* both include 36 genes, containing 12 PCGs (*cox1-3*, *nad1-6*, *nad4L*, *cytb*, and *atp6*; missing *atp8*), 22 transfer RNA (tRNA) genes, and two ribosomal RNAs (*rrnS* and *rrnL*), plus two noncoding regions (SNCR is 160 bp, located between *trnQ* and *trnY* in *M. hemirhamphi *versus SNCR, which is 244 bp, between *trnK* and *trnE* in *R. laterospinosus*; LNCR is 4,238 bp, located between *trnK* and *trnV* in *M. hemirhamphi* versus LNCR, which is only 252 bp, located between *trnQ* and *trnY* in *R. laterospinosus*) (Fig. 2, Table 4). All mitochondrial genes are encoded on the same strand in the same direction. The lengths of the complete mitogenomes of *M. hemirhamphi* and *R. laterospinosus* are 17,272 bp and 13,567 bp, respectively. The overall A + T contents of *M. hemirhamphi* and *R. laterospinosus* mitogenomes are 60.5% and 62.9%, which both have a strong A + T bias. The nucleotide contents of the mitogenomes of *M. hemirhamphi* and *R. laterospinosus* are provided in Tables 4 and 5.

**Table 4:** Organization of the mitochondrial genomes of *Micracanthorhynchina hemirhamphi* and *Rhadinorhynchus laterospinosus*

*Micracanthorhynchina hemirhamphi*						*Rhadinorhynchus laterospinosus*					
Gene/region	Position 5′ to 3′	Size (bp)	Ini/Ter cod	Anticodon	Int seq	Gene/region	Position 5′ to 3′	Size (bp)	Ini/Ter cod	Anticodon	Int seq
*cox1*	1–1,536	1,536	GTG/TAA		0	*cox1*	1–1,539	1,539	GTG/TAG		−2
*trnY*	1537–1588	52		gua	0	*trnG*	1,538–1,590	53		ucc	−11
*SNCR*	1,589–1,748	160			0	*trnQ*	1,580–1,635	56		uug	0
*trnQ*	1,749–1,810	62		uug	47	*LNCR*	1,636–1,887	252			0
*trnG*	1,858–1,909	52		ucc	0	*trnY*	1,888–1,938	51		gua	0
*rrnL*	1,910–2,826	917			0	*rrnL*	1,939–2,854	916			0
*trnL1*	2,827–2,877	51		uag	0	*trnL1*	2,855–2,906	52		uag	0
*nad6*	2,878–3,327	450	GTG/TAA		−2	*nad6*	2,907–3,335	429	GTG/TAG		95
*trnD*	3,326–3,377	52		auc	9	*trnD*	3,431–3,483	53		guc	12
*trnS2*	3,387–3,437	51		uga	15	*atp6*	3,496–4,008	513	ATG/TAG		−5
*atp6*	3,453–3,959	507	GTG/TAG		1	*nad3*	4,004–4,343	340	ATT/T		0
*nad3*	3,961–4,363	403	GTT/T		0	*trnW*	4,344–4,409	66		uca	130
*trnW*	4,364–4,417	54		uca	108	*trnK*	4,540–4,598	59		uuu	0
*trnK*	4,526–4,584	59		uuu	0	*SNCR*	4,599–4,842	244			0
*LNCR*	4,585–8,822	4,238			0	*trnE*	4,843–4,894	52		uuc	−6
*trnV*	8,823–8,884	62		uac	−13	*trnT*	4,889–4,942	54		ugu	−24
*trnE*	8,872–8,922	51		uuc	−7	*trnS2*	4,919–4,979	61		uga	12
*trnT*	8,916–8,971	56		ugu	7	*nad4L*	4,992–5,231	240	TTG/TAA		−7
*nad4L*	8,979–9,240	262	TTG/T		12	*nad4*	5,225–6,420	1196	GTG/TA		0
*nad4*	9,253–10,456	1204	GTG/T		−2	*trnV*	6,421–6,484	64		uac	190
*trnH*	10,455–10,508	54		gug	−1	*trnH*	6,675–6,729	55		gug	0
*nad5*	10,508–12,122	1615	ATA/T		0	*nad5*	6,730–8,346	1617	GTG/TAG		−1
*trnL2*	12,123–12,174	52		uaa	−10	*trnL2*	8,346–8,397	52		uaa	0
*trnP*	12,165–12,217	53		ugg	0	*trnP*	8,398–8,448	51		ugg	0
*cytb*	12,218–13,325	1,108	ATA/T		3	*cytb*	8,449–9,565	1,117	ATG/T		−2
*nad1*	13,329–14,211	883	ATG/T		−1	*nad1*	9,564–10,443	880	ATG/T		0
*trnI*	14,211–14,263	53		gau	−2	*trnI*	10,444–10,502	59		gau	−12
*trnM*	14,262–14,311	50		cau	0	*trnM*	10,491–10548	58		cau	0
*rrnS*	14,312–14,825	514			0	*rrnS*	10,549–11,084	536			0
*trnF*	14,826–14,885	60		gaa	0	*trnF*	11,085–11,139	55		gaa	2
*cox2*	14,886–15,504	619	GTG/T		−1	*cox2*	11,142–11,765	624	ATG/TAA		−2
*trnC*	15,504–15,554	51		gca	1	*trnC*	11,764–11,816	53		gca	15
*cox3*	15,556–16,236	681	GTG/TAA		−2	*cox3*	11,832–12,509	678	GTG/TAA		−2
*trnA*	16,235–16,287	53		ugc	−7	*trnA*	12,508–12,562	55		ugc	−8
*trnR*	16,281–16,332	52		ucg	−14	*trnR*	12,555–12,615	61		ucg	−16
*trnN*	16,319–16,374	56		guu	−19	*trnN*	12,600–12,655	56		guu	−17
*trnS1*	16,356–16,414	59		acu	2	*trnS1*	12,639–12,696	58		acu	0
*nad2*	16,417–17,271	855	GTG/TAA		1	*nad2*	12,697–13,564	868	TTG/T		1

“Ini/Ter cod” and “Int seq” indicate initial/terminal codons and the length of intergenic sequences, respectively

**Table 5:** Base composition and skewness in the mitogenomes of *Micracanthorhynchina hemirhamphi* and* Rhadinorhynchus laterospinosus*

Location/species	A%	T%	C%	G%	AT%	AT-skew	GC-skew	Total (bp)
*M. hemirhamphi*								
Whole mitochondrial genome	22.46	38.06	10.01	29.46	60.53	−0.26	0.49	17,272
Protein coding genes (PCGs)	20.15	38.23	10.16	31.46	58.38	−0.31	0.51	10,123
First codon	21.34	31.49	9.88	37.29	52.83	−0.19	0.58	3,379
Second codon	12.75	47.98	11.86	27.40	60.74	−0.58	0.40	3,372
Third codon	26.36	35.23	8.72	29.69	61.60	−0.14	0.55	3,372
tRNAs	28.28	37.99	9.46	24.27	66.28	−0.15	0.44	1,195
rRNAs	28.72	34.10	11.95	25.23	62.82	−0.09	0.36	1,431
*rrnS*	29.38	32.68	11.67	26.26	62.06	−0.05	0.38	514
*rrnL*	28.35	34.90	12.10	24.65	63.25	−0.10	0.34	917
LNCR	24.26	39.48	8.75	27.51	63.74	−0.24	0.52	4,238
SCNR	22.50	34.38	17.50	25.62	56.88	−0.21	0.19	160
*R. laterospinosus*								
Whole mitochondrial genome	24.21	38.67	10.69	26.43	62.88	−0.23	0.42	13,567
Protein coding genes (PCGs)	22.23	38.98	10.86	27.94	61.21	−0.27	0.44	10,041
First codon	23.88	29.76	10.42	35.94	53.64	−0.11	0.55	3,350
Second codon	13.75	48.03	13.00	25.22	61.78	−0.55	0.32	3,346
Third codon	29.06	39.16	9.15	22.63	68.22	−0.15	0.42	3,345
tRNAs	27.55	38.17	10.62	23.66	65.72	−0.16	0.38	1,234
rRNAs	32.16	35.19	10.74	21.90	67.36	−0.04	0.34	1,452
*rrnS*	32.09	34.33	11.01	22.57	66.42	−0.03	0.34	536
*rrnL*	32.21	35.70	10.59	21.51	67.90	−0.05	0.34	916
LNCR	26.59	48.01	6.75	18.65	74.60	−0.29	0.47	252
SCNR	35.25	37.30	6.15	21.30	72.55	−0.03	0.55	244

**Fig. 3:**
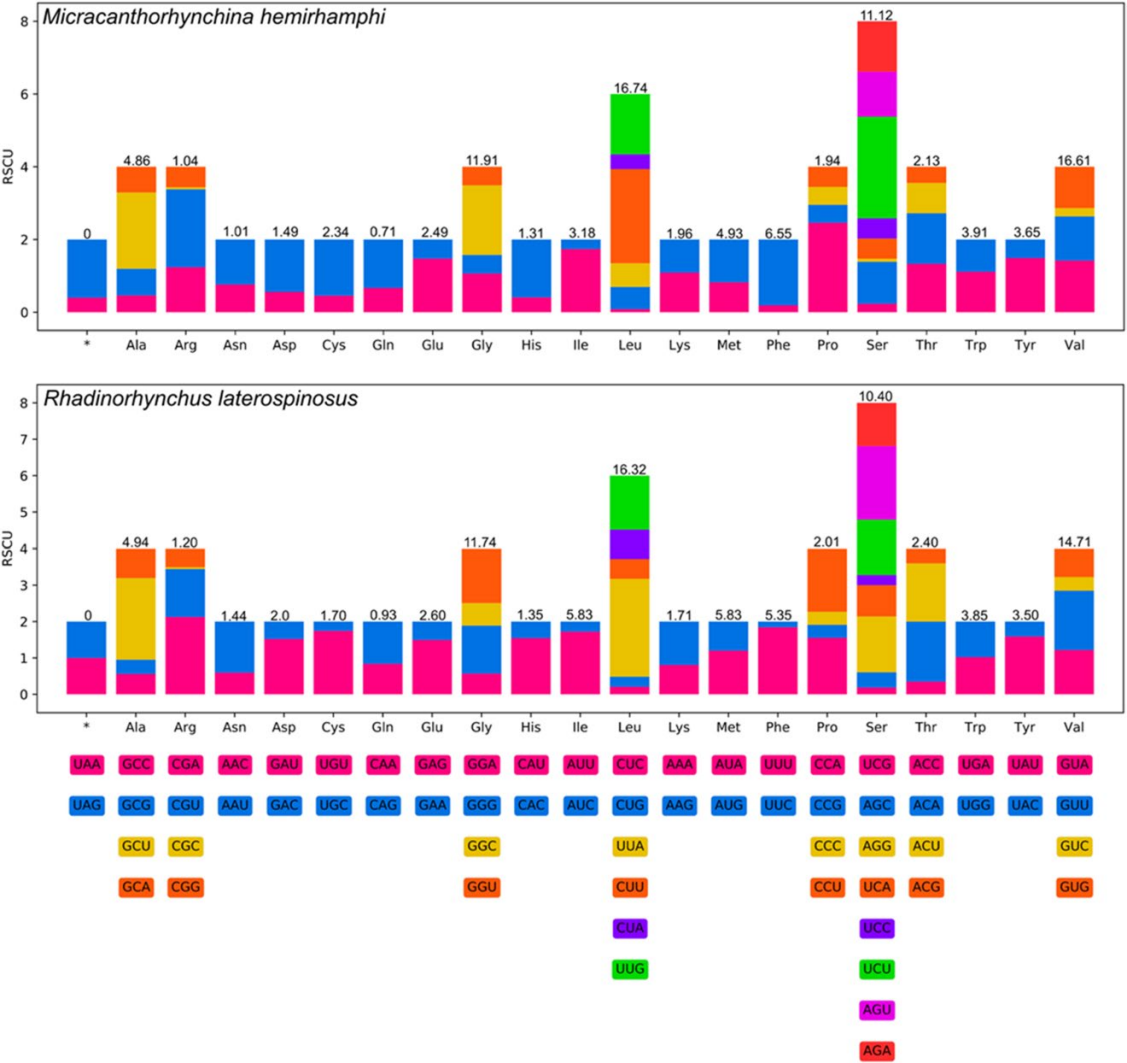
Relative synonymous codon usage (RSCU) of *Micracanthorhynchina hemirhamphi* and *Rhadinorhynchus laterospinosus.* The codon families (in alphabetical order) are labeled on the *x*-axis. Values on the top of each bar represent amino acid usage in percentage

The 12 PCGs in the mitogenomes of *M. hemirhamphi* and *R. laterospinosus* have 10,123 bp and 10,041 bp (excluding termination codons), and encode 3,372 and 3,345 amino acids, respectively. The size of each of the 12 PCGs in the mitogenomes of *M. hemirhamphi* and *R. laterospinosus* are provided in Table 5. Among the 12 PCGs of *M. hemirhamphi*, seven genes (*cox1*, *cox2*, *cox3*, *nad2*, *nad4*, *nad6*, and *atp6*) use GTG as the start codon, followed by two genes (*cytb* and *nad5*) that use ATA, while *nad1*, *nad3*, and *nad4L* use ATG, GTT, and TTG as the start codon, respectively. A total of four genes (*cox1*, *cox3*, *nad2*, and *nad6*) use TAA as the termination codon, and only *atp6* uses TAG; the remaining seven genes (*nad1*, *nad3*, *nad4*, *nad4L*, *nad5*, *cox2* and *cytb*) are inferred to terminate with incomplete stop codon T (Table 4). Among the 12 PCGs of *R. laterospinosus*, five genes (*cox1*, *cox3*, *nad4*, *nad5*, and *nad6*) use GTG as the start codon, followed by four genes (*atp6*, *cytb*, *nad1*, and *cox2*) that use ATG, while two genes (*nad2* and *nad4L*) use TTG, and only *nad3* uses ATT as the start codon. A total of four genes (*cox1*, *nad5*, *nad6*, and *atp6*) use TAG as the termination codon, followed by three genes (*cox2*, *cox3*, and *nad4L*) that use TAA, while only *nad4* uses the incomplete stop codon TA. The remaining four genes (*nad1*, *nad2*, *nad3*, and *cytb*) are inferred to terminate with an incomplete stop codon T (Table 4). The codon usage in the mitogenomes of *M. hemirhamphi* and *R. laterospinosus* is presented in Fig. 3.

The lengths of 22 tRNAs in the mitogenomes of *M. hemirhamphi* and *R. laterospinosus* are from 50 to 66 bases (Table 2). Their anticodon secondary structures are provided in Supplementary Material Figs. S1 and S2. In the mitogenomes of *M. hemirhamphi* and *R. laterospinosus*, the small ribosomal RNA gene (*rrnS*) is located between *trnM* and *trnF* in both species, with 514 bp to 536 bp in length, respectively; while the large ribosomal RNA gene (*rrnL*) is located in a different position with similar length in the two species (917 bp long, between *trnG* and *trnL1* in *M. hemirhamphi, versus* 916 bp long, between *trnY* and *trnL1* in *R. laterospinosus*).

**Fig. 4:**
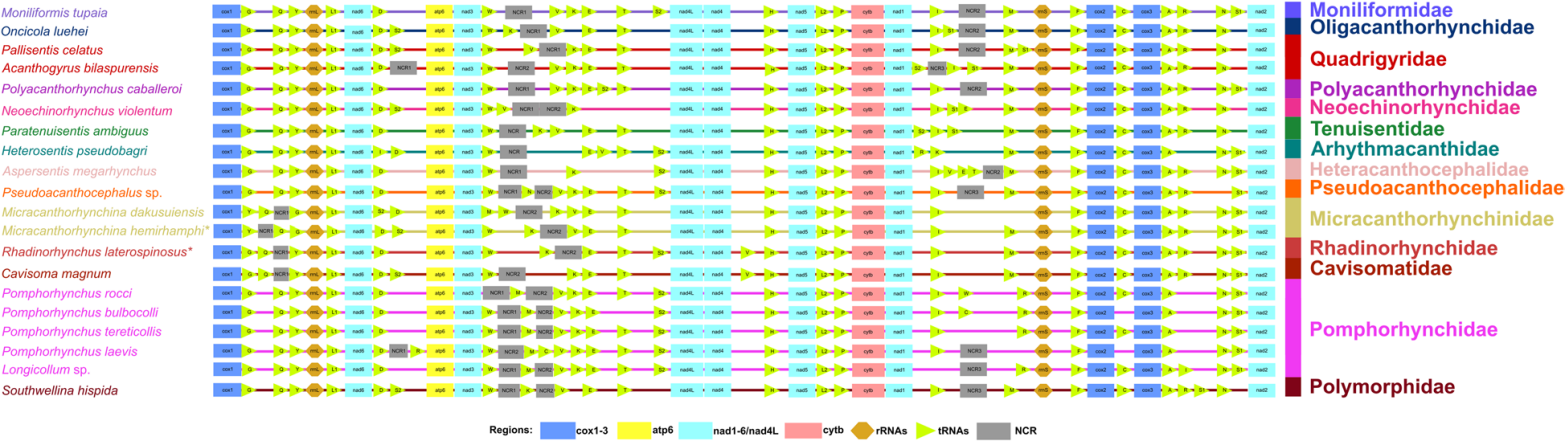
Comparison of the linearized mitochondrial genome arrangement for acanthocephalans species (a total of 18 types of 36 gene arrangement in the mitogenomes of Acanthocephala reported before this study). All genes are transcribed in the same direction from left to right. The tRNAs are labelled by a single-letter code for the corresponding amino acid. *Micracanthorhynchina hemirhamphi* and *Rhadinorhynchus laterospinosus* are indicated using asterisk (*)

In the mitogenomes of *M. hemirhamphi* and *R. laterospinosus*, the gene arrangements of 12 PCGs and two rRNAs are in the typical order of acanthocephalans: *cox1*, *rrnL*, *nad6*, *atp6*, *nad3*, *nad4L*, *nad4*, *nad5*, *cytb*, *nad1*, *rrnS*, *cox2*, *cox3*, and *nad2*. However, translocations of several tRNAs occurred in both mitogenomes of *M. hemirhamphi* and *R. laterospinosus* (Figs. 2 and 4).

### Phylogenetic analyses

**Fig. 5:**
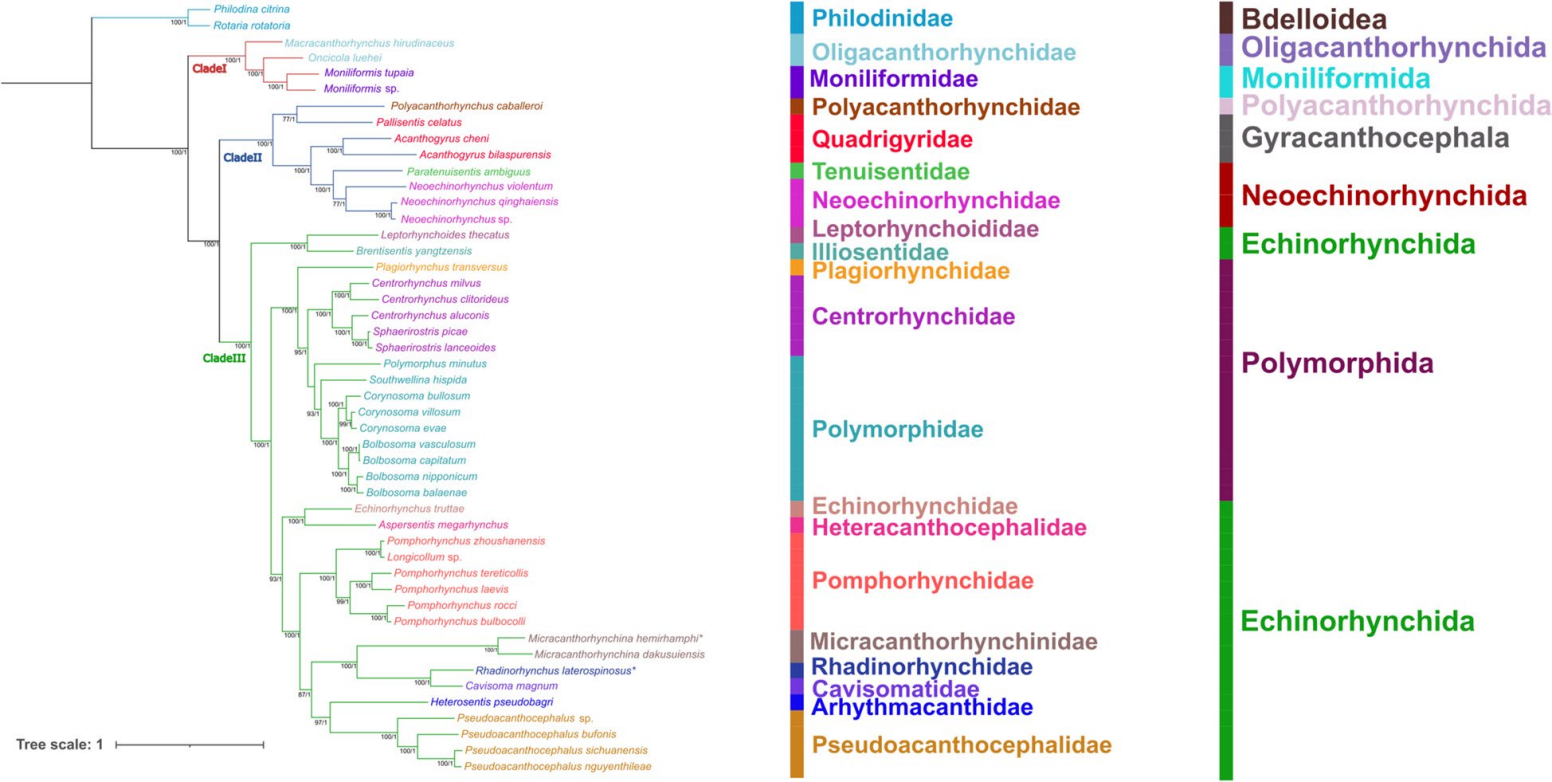
Phylogenetic results using ML and BI methods based on the concatenating amino acid sequences of 12 protein-coding genes (PCGs) of acanthocephalan mitogenomes. *Rotaria rotatoria* and *Philodina citrina* are treated as the out-group. Bootstrap values ≥ 75 and Bayesian posterior probabilities values ≥ 0.95 are shown in the phylogenetic tree. *Micracanthorhynchina hemirhamphi* and *Rhadinorhynchus laterospinosus* are indicated using asterisk (*)

The topologies of phylogenetic trees based on the amino acid sequences of 12PCGs using ML and BI methods, are nearly identical, which both support the division of the phylum Acanthocephala into three major monophyletic clades (clades I, II, and III) (Fig. 5). Clade I includes *Macracanthorhynchus hirudinaceus*, *Oncicola luehei, Moniliformis tupaia*, and *Moniliformis* sp., representing the class Archiacanthocephala. Clade II includes species of the orders Gyracanthocephala and Neoechinorhynchida, belonging to the class Eoacanthocephala, together with *Polyacanthorhynchus caballeroi* (a member of the class Polyacanthocephala). Clade III contains representatives of the orders Echinorhynchida and Polymorphida, representing the class Palaeacanthocephala. *Micracanthorhynchina hemirhamphi* and *M. dakusuiensis* clustered together, representing the family Micracanthorhynchinidae. The rhadinorhynchid species *R. laterospinosus* formed a sister relationship with a member of the Cavisomatidae (*Cavisoma magnum*) with maximum support.

## Discussion

To date, a total of 17 acanthocephalan species representing nine different families within the order Echinorhynchida have been sequenced for their mitogenomes [11, 17, 21, 22, 26, 34, 41]. However, in the Rhadinorhynchidae, only *Micracanthorhynchina dakusuiensis* belonging to the subfamily Gorgorhynchinae has had the complete mitogenome reported so far [26]. This study provided the mitogenomes of *M. hemirhamphi* and *R. laterospinosus* for the first time. The mitogenome of *R. laterospinosus* represents the first mitogenomic data for the genus *Rhadinorhynchus* and also for the subfamily Rhadinorhynchinae.

The mitogenome of *M. dakusuiensis* has 16,309 bp, which is the largest in the known mitogenome of Acanthocephala. However, comparative mitogenomic analysis showed that the mitogenome of *M. hemirhamphi* has 17,272 bp and is distinctly larger than that of *M. dakusuiensis* [26]. The mitogenomes of *M. hemirhamphi* and *M. dakusuiensis* are rather large because the LNCRs are very long in their mitogenomes. Additionally, the complete mitogenome of *R. laterospinosus* (13,567 bp) represents the smallest one in the reported mitogenomes of Echinorhynchida so far. The overall A + T contents in the mitogenomes of *M. hemirhamphi* (60.5%) and *R. laterospinosus* (62.9%) are similar to that of the known Echinorhynchida mitogenomes [55.8% (*Pomphorhynchus zhoushanensis*) to 71.4% (*Leptorhynchoides thecatus*)].

The organization and arrangements of the 12 PCGs and two rRNAs in the reported mitogenomes of Acanthocephala are rather conserved, which are all in the typical order (*cox1*, *rrnL*, *nad6*, *atp6*, *nad3*, *nad4L*, *nad4*, *nad5*, *cytb*, *nad1*, *rrnS*, *cox2*, *cox3*, and *nad2*), except for *Echinorhynchus truttae*, lacking *nad4* and *nad4L* (the authors failed to sequenced the *nad4* and *nad4L*) [34]. By contrast, the positions of 22 tRNAs seem to exhibit a high degree of variation among different groups [12, 14, 21, 24, 26, 35]. To date, a total of 18 types of gene arrangements of 22 tRNAs in the mitogenomes of Acanthocephala have been found [24]. The present study revealed that the gene arrangement in the mitogenomes of *M. hemirhamphi* and *R. laterospinosus* are different from that of the known mitogenomes of Acanthocephala.

Recently, some phylogenetic studies indicated that the Rhadinorhynchidae is a non-monophyletic group based on the 18S + 28S, 18S + *cox1*, or 18S + 28S + *cox1* datasets, which challenged and improved the current classifications of the Rhadinorhynchidae [8–10, 26, 28, 51, 52]. For example, the genus *Gorgorhynchoides* belonging to the subfamily Gorgorhynchinae and the genus *Serrasentis* belonging to the subfamily Serrasentinae in the traditional classification of the Rhadinorhynchidae, were recently transferred into the family Isthmosacanthidae in the order Polymorphida [8]. Kita et al. [10] suggested the resurrection of the validity of the family Micracanthorhynchinidae based on the phylogenetic analyses of 18S + 28S + *cox1* data. The present mitogenomic phylogenetic results are accordant with a previous study [10], which also supported the proposal of resurrection of Micracanthorhynchinidae to resolve the non-monophyly of the Rhadinorhynchidae. *Micracanthorhynchina* spp. have a short proboscis with a small number of hooks in the longitudinal row and four or six short claviform or club-shaped cement glands clustered together, which are different from that of the other rhadiorhynchid groups.

The previous study based on the 18S + 28S + *cox1* sequence data showed the Micracanthorhynchinidae is a sister to Transvenidae + Rhadinorhynchidae sensu stricto + Cavisomatidae within Echinorhynchida [10]. However, the present mitogenomic phylogenis did not include Transvenidae due to the mitogenomic data of transvenid species being unavailable. Our results revealed a close affinity between Rhadinorhynchidae sensu stricto and Cavisomatidae, which formed a sister relationship with the Micracanthorhynchinidae (Micracanthorhynchinidae + (Rhadinorhynchidae sensu stricto + Cavisomatidae)). A more rigorous mitogenomic phylogeny including broader taxon sampling of Echinorhynchida, especially the key families and subfamilies currently lacking genetic data, is needed to further clarify the evolutionary relationships of the different families and subfamilies within Echinorhynchida.

## Conclusions

The complete mitogenomes of *M. hemirhamphi* and *R. laterospinosus* are provided for the first time, which both include 36 genes, containing 12 PCGs (missing *atp8*), 22 tRNA genes, and two ribosomal RNAs (*rrnS* and *rrnL*), plus two noncoding regions. The mitogenome of *M. hemirhamphi* has 17,272 bp and represents the largest mitogenome of acanthocephalan reported so far. The mitogenome of *R. laterospinosus* has only 13,567 bp and is the smallest mitogenome of the order Echinorhynchida, which also represents the first mitogenomic data for the Rhadinorhynchidae* sensu stricto*. Several tRNA gene rearrangement events occur in the mitogenomes of both *R. laterospinosus* and *M. hemirhamphi*, which represent two new types of the gene arrangement in the mitogenomes of Acanthocephala. Moreover, phylogenetic analyses based on the amino acid sequences of 12 protein-coding genes of mitogenomes further confirmed the validity of the family Micracanthorhynchinidae and suggested a sister relationship Micracanthorhynchinidae + (Rhadinorhynchidae + Cavisomatidae) within Echinorhynchida.

## Supplementary Information

Additional file 1 (Fig. S1. The predicted secondary structures of 22 tRNAs in the mitogenome of Micracanthorhynchina hemirhamphi (Watson-Crick bonds indicated by lines, GU bonds indicated by dots, grey bold bases representing anticodons). The tRNAs were labelled with the abbreviations of their corresponding amino acids according to the IUPAC-IUB code.)

Additional file 2 (Fig. S2. The predicted secondary structures of 22 tRNAs in the mitogenome of Rhadinorhynchus laterospinosus (Watson-Crick bonds indicated by lines, GU bonds indicated by dots, grey bold bases representing anticodons). The tRNAs were labelled with the abbreviations of their corresponding amino acids according to the IUPAC-IUB code.)

## Data Availability

Voucher specimens of *M. hemirhamphi* and *R. laterospinosus* were deposited in the College of Life Sciences, Hebei Normal University, China. Sequence data that support the findings of this study have been deposited in the National Center for Biotechnology Information (NCBI) database (https://www.ncbi.nlm.nih.gov) with the accession numbers: PV590120 (*M. hemirhamphi*) and PV590110 (*R. laterospinosus*).
