# Supplementary figures and images for: Mitogenomic phylogenies support the validity of the family Micracanthorhynchinidae (Acanthocephala: Echinorhynchida), with novel gene arrangement in the mitogenomes of Micracanthorhynchina hemirhamphi and Rhadinorhynchus laterospinosus
Source: Parasit Vectors. 2025 Aug 3;18:328. doi: 10.1186/s13071-025-06972-z (PMC12318431; doi:10.1186/s13071-025-06972-z)

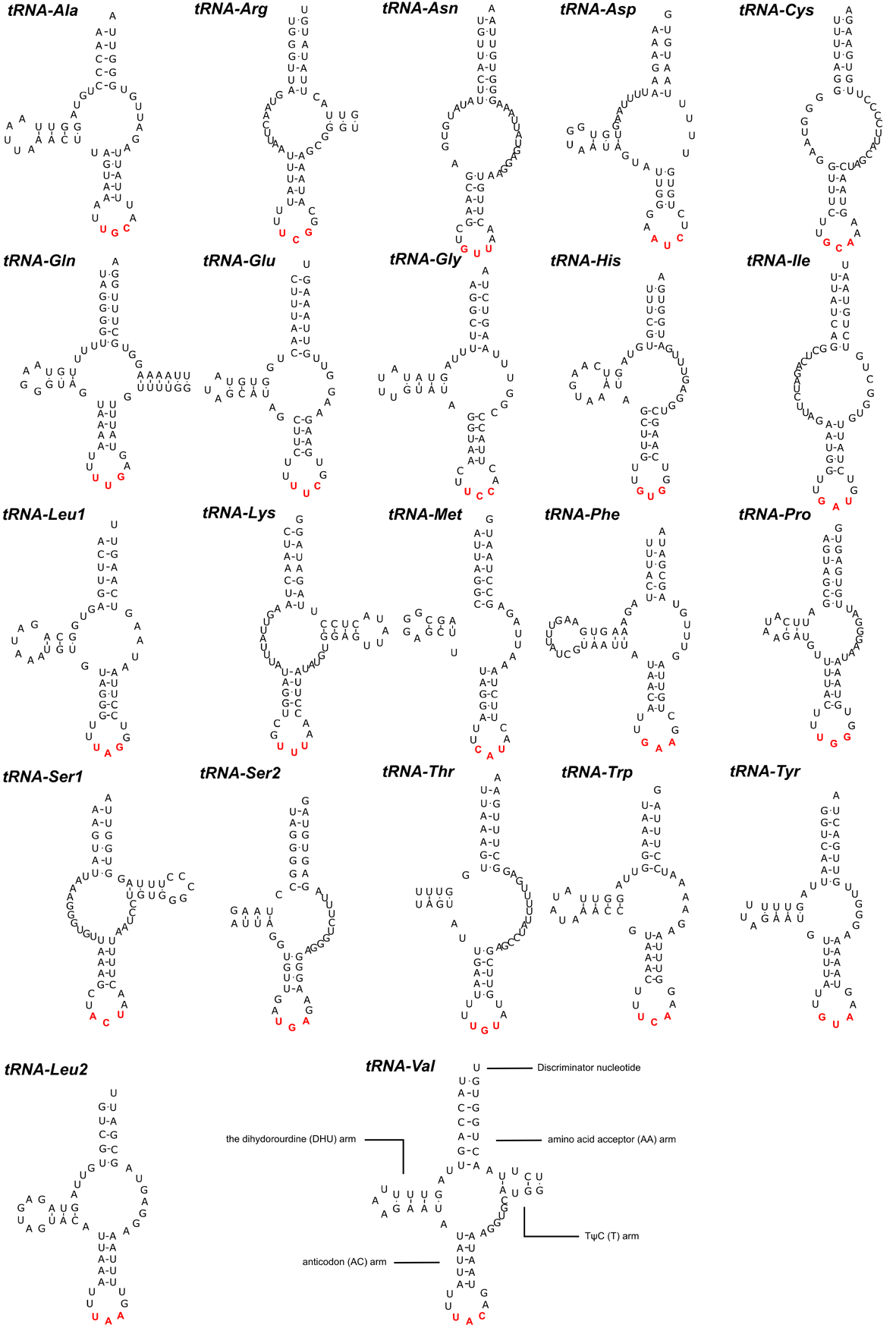

Supplement: Supplementary file 1 — Additional file 1 (Fig. S1. The predicted secondary structures of 22 tRNAs in the mitogenome of Micracanthorhynchina hemirhamphi (Watson-Crick bonds indicated by lines, GU bonds indicated by dots, grey bold bases representing anticodons). The tRNAs were labelled with the abbreviations of their corresponding amino acids according to the IUPAC-IUB code.) [file 13071_2025_6972_MOESM1_ESM.tiff]

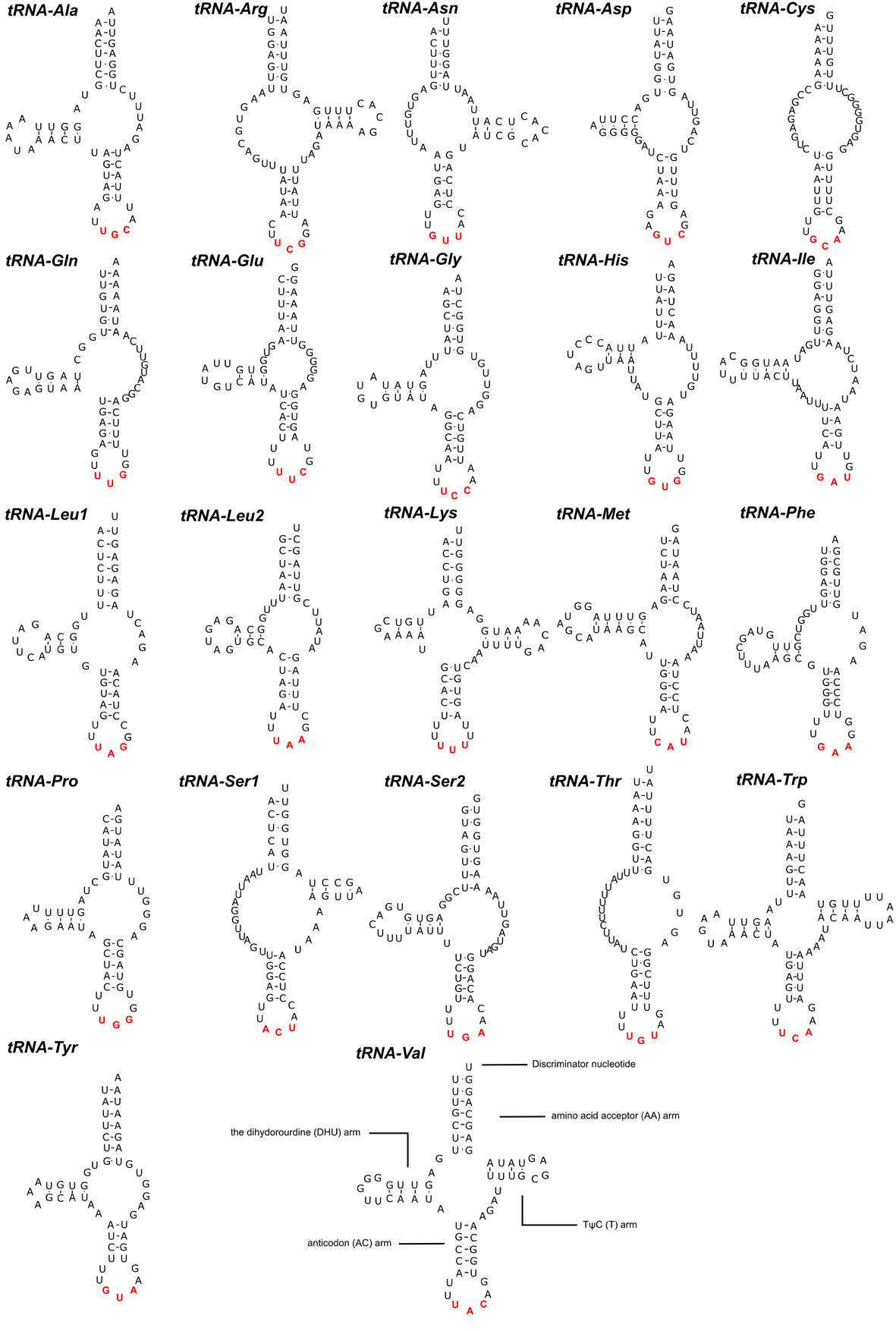

Supplement: Supplementary file 2 — Additional file 2 (Fig. S2. The predicted secondary structures of 22 tRNAs in the mitogenome of Rhadinorhynchus laterospinosus (Watson-Crick bonds indicated by lines, GU bonds indicated by dots, grey bold bases representing anticodons). The tRNAs were labelled with the abbreviations of their corresponding amino acids according to the IUPAC-IUB code.) [file 13071_2025_6972_MOESM2_ESM.tiff]
